# Regional citrate anticoagulation for continuous renal replacement therapy in newborns

**DOI:** 10.3389/fped.2023.1089849

**Published:** 2023-03-08

**Authors:** Haixia Huang, Xing Deng, Ke Bai, Chengjun Liu, Feng Xu, Hongxing Dang

**Affiliations:** Intensive Care Unit, Ministry of Education Key Laboratory of Children Development and Disorders, China International Science and Technology Cooperation Base of Child Development and Critical Disorders, Chongqing Key Laboratory of Pediatrics, Children’s Hospital of Chongqing Medical University, Chongqing, China

**Keywords:** citrate accumulation, continuous renal replacement therapy, newborns, regional citrate anticoagulation, critically ill

## Abstract

**Background:**

Regional citrate anticoagulant (RCA) is recommended as the preferred anticoagulant regimen for continuous renal replacement therapy (CRRT) in adults; however, it is rarely reported in neonates due to concerns associated with their immature liver. Few studies have reported on the use of RCA to evaluate the safety and efficacy of RCA-CRRT in neonates.

**Method:**

In this retrospective observational study, we reviewed the clinical records of neonates who underwent RCA-CRRT at our pediatric intensive care unit between September 2015 to January 2021.

**Results:**

A total of 23 neonates underwent 57 sessions of RCA-CRRT. Their mean age was 10.1 ± 6.9 days and mean weight was 3.0 ± 0.7 kg (range, 0.95–4 kg). The mean filter life was 31.54 ± 19.58 h (range, 3.3–72.5 h). Compared to pretreatment values, the total-to-ionized calcium ratio (T/iCa) on RCA-CRRT increased (2.00 ± 34 0.36 vs. 2.19 ± 0.40, *P* = 0.056) as did the incidence of T/iCa levels >2.5 (11.4 vs. 14.3, *P* = 0.477), albeit not significantly. Using a post-treatment T/iCa threshold of 2.5, we divided all the cases into citrate accumulation (CA) and non-CA (NCA) groups. Compared with the NCA group, the CA group had significantly higher body weight (3.64 ± 0.32 kg vs. 2.95 ± 0.41 kg, *P* = 0.033) and significantly lower blood flow rate per body weight ml/kg/min (3.08 ± 0.08 vs. 4.07 ± 0.71, *P* = 0.027); however, there was no significant difference between the two groups in terms of age, corrected gestational age, the PRISM-III score, and biochemical tests.

**Conclusion:**

RCA-CRRT is safe and effective for neonates. After appropriate adjustments of the RCA-CRRT parameters, the incidence of CA was not higher in neonates than in children or adults, and CA was not found to be significantly correlated with age or corrected gestational age.

## Introduction

Continuous renal replacement therapy (CRRT) increases the chances of survival of critically ill patients by preventing fluid overload, correcting the acid–base status and electrolyte imbalance, and allowing optimal use of parenteral nutrition (1). With advances in neonatal care and CRRT technology, CRRT is being increasingly applied in NICU for treating acute kidney injury (AKI) (2, 3), multiorgan dysfunction, and metabolic diseases, such as hyperammonemia (4). Neonatal CRRT is the most technically demanding CRRT of any age group. The major challenges associated with neonatal CRRT use are related to the establishment of vascular access and the management of anticoagulation and hemodynamics. The current reports on neonatal CRRT mainly concern systemic heparin anticoagulation (5) and no heparin anticoagulation (6, 7), and RCA has only been reported in individual cases (8–10). With CRRT, the risk of clotting is higher in neonates than in children and adults due to the slow blood flow. In addition, critically ill neonates often have coagulation disorders, and premature neonates are prone to intracranial hemorrhage and pulmonary hemorrhage. Therefore, anticoagulation during CRRT in neonates is a major clinical challenge. The biggest advantage of RCA is that it is fully anticoagulated *in vitro* and has no effect on coagulation *in vivo*. RCA was recommended by KDIGO in 2012 as the preferred anticoagulation modality for CRRT in cases/patients without contraindications to citrate anticoagulation (11). In recent years, studies on RCA-CRRT have been increasingly reported in children (12, 13) but are still rare in neonates. This could be attributed to neonatologists’ concerns about the immaturity of the neonatal liver, which affects neonates’ ability to metabolize citrate and leads to RCA-related complications. In addition, neonatal RCA-CRRT regimens may differ from adults and even children, such as in calcium supplementation, because newborns are at greater risk of hypocalcemia due to immature sarcoplasmic reticulum development. Although neonatal RCA-CRRT has also been reported individually in the form of co-reports in small infants, to our knowledge, RCA-CRRT has not been reported exclusively in neonates (14). Therefore, such reports cannot reflect the differences between neonates and children or adults undergoing RCA-CRRT.

The study aims to share our experience with RCA-CRRT in neonates, especially concerning the technical and practical aspects of such treatment.

## Methods

### Patients

We conducted a retrospective observational study of newborns (age <28 days, irrespective of gestational age) treated with RCA-CRRT in the pediatric intensive care unit at the Children's Hospital of Chongqing Medical University from September 2015 to January 2021. The study was approved by the ethical committee of Children's Hospital of Chongqing Medical University (approval no.: 2022.120).

### CRRT procedures

The PlasautoΣ blood purification device (Asahi Kasei Kuraray Medical, Japan) was used for all patients with AEF-03 filters (filter capacity: 26 ml) and CRRT-CSGNL1 tubes (capacity: 47 ml). Vascular access for CRRT was a 5F double-lumen catheter (ARROW: CS-14502) inserted through the right or left internal jugular vein, or one 5F double-lumen catheter each through the right or left internal jugular vein, or a 20–22G indent needle through the brachial or femoral artery. All CRRT procedures were performed as continuous veno-venous hemodiafiltration. After routine priming the CRRT cycle with heparin saline and saline, 0.25 U red blood cell suspension and 5 g of 20% albumin stock solution were given to prime the CRRT cycle. Calcium-free treatment solutions for dialysis and replacement were self-configured with the following concentrations: ionized sodium, 113 mmol/L; iCa^2+^, 1.6 mmol/L; Mg^2+^, 0.797 mmol/L; Cl^−^, 118 mmol/L; and anhydrous glucose, 10.6 mmol/L. The citrate sodium anticoagulant (4%; approval no.: H20058913; Sichuan Nightingale Biological, China) was infused into the extracorporeal blood circulation using an infusion pump (Optima PT, Fresenius SE & Co. KGaA, Germany) through a T-junction connected at the primer of the artery port. Calcium gluconate (10%) was infused into the extracorporeal blood circulation by an Agilia infusion micropump (Fresenius SE & Co. KGaA, Germany) through a T-junction connected at the end of the venous port.

The initial parameters were set as shown in Table 1. First, we adjusted the flow rate of 4% sodium citrate (CiFR) to reach the target concentration of ionized calcium *in vitro* of 0.2–0.4 mmol/L. If ionized calcium increased *in vitro*, we increased CiFR, and vice versa. Then, we adjusted the flow rate of 10% calcium gluconate (CaFR) to reach the target ionized calcium *in vivo* concentration of 1.0–1.35 mmol/L. If ionized calcium increased *in vivo*, we decreased CaFR, and vice versa. Finally, we adjusted sodium bicarbonate 5% rate (SBFR) according to blood gas analysis to reach a target bicarbonate concentration of 22–27 mmol/L.

**Table 1:** Comparison of initial and final parameters.

	Initial parameter reference	Initial parameters	Final parameters	*P* value
BFR/BW, ml/kg/min	3–5	3.82 ± 0.86	3.92 ± 0.87	0.078
CiFR/(60*BFR)	1.5	1.41 ± 0.17	1.48 ± 0.17	0.014
Moles of citrate per Molers of citrate per CiFR/BW, mmol/kg/h	0.61–1.02	0.70 (0.62, 0.86)	0.74 (0.64, 0.94)	0.110
CaFR/(60*BFR)	0.1	0.15 ± 0.06	0.17 ± 0.07	0.014
DFR/BW, ml/kg/h	25	21.74 (16.67, 30.30)	25.86 (18.63, 34.48)	0.302
FFR/BW, ml/kg/h	35	30.30 (23.33, 33.65)	30.43 (24.19, 36.52)	0.166
(DFR + FFR)/BW, ml/kg/h	60	52.17 (40.00, 64.04)	59.57 (45.00, 69.27)	0.236
SBFR/(DFR + FFR)	0.01	0.011 (0.008, 0.017)	0.012 (0.000, 0.020)	0.643

BFR, blood flow rate, ml/min; BW, body weight, kg; CiFR, flow rate of 4% sodium citrate, ml/h; CaFR, flow rate of 10% calcium gluconate, ml/h; DFR, dialysate flow rate, ml/h; FFR, filtrate flow rate, ml/h; SBFR, 5% sodium bicarbonate flow rate, ml/h. Considering a newborn weighing 3 kg as an example, the initial parameters were set as BFR = 10 ml/min, CiFR = 15 ml/h, CaFR = 1 ml/h, DFR = 75 ml/h, FFR = 105 ml/h, and SBFR = 1.8 ml/h.

Blood gas analysis (ABL90 FLEX Analyzer), both *in vivo* and *in vitro*, was conducted every 30 min after starting the treatment until the *in vitro* target of ionized calcium was achieved, and then, it was routinely performed every 4–6 h. Biochemical indicators and electrolytes were checked every 12–24 h.

### Data collection and definition

**Figure 1:**
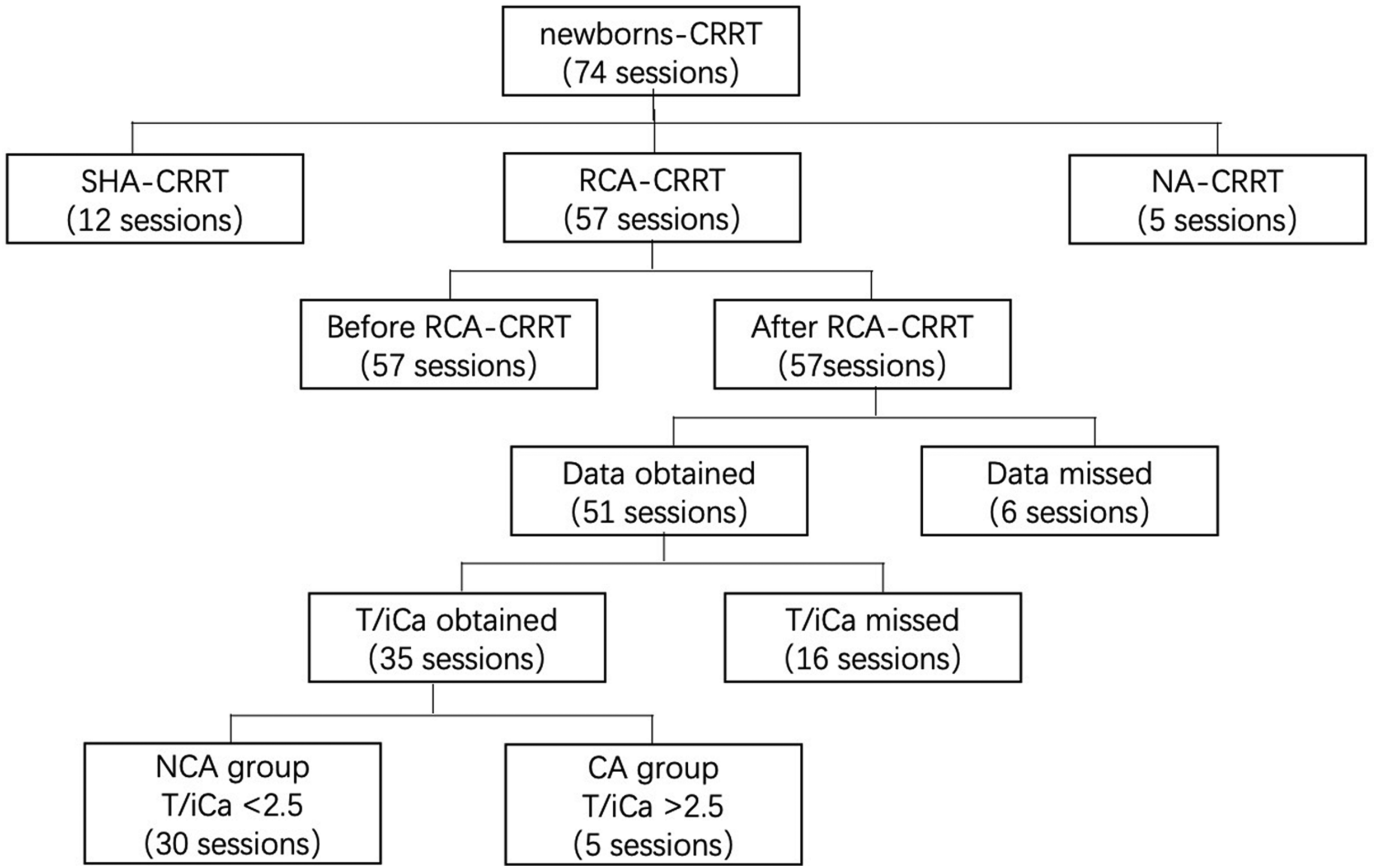
Data collection. CRRT, continuous renal replacement therapy; SHA, systemic heparin anticoagulation; NA, no anticoagulation; CA, citrate accumulation, T/iCa > 2.5 at the end of RCA-CRRT; NCA, no citrate accumulation, T/iCa < 2.5 at the end of RCA-CRRT; T/iCa, ratio of total calcium to ionic calcium.

Data on the CRRT operating parameters, filter lifespan, reasons for CRRT, and laboratory results were collected from medical records. We included cases wherein the treatment administered was CRRT anticoagulated with RCA in newborns (age, <28 days). We excluded cases wherein the treatment administered was CRRT anticoagulated with heparin or CRRT without anticoagulation. The indications of CRRT were: (i) Neonatal AKI with fluid overload, or electrolyte disorder or internal environment improved not obviously after regular treatment (15); (ii) Hyperaminoemia. blood ammonia level >150 μmol/L with rapidly deteriorating neurological status, coma, or cerebral oedema after regular treatment (16). Data characteristics are shown in Figure 1.

Citrate accumulation (CA) was defined as the ratio of total-to-ionized calcium (T/iCa) being >2.5. Patients were divided into CA and non-CA (NCA) groups according to whether their condition was complicated with CA after treatment. Hypocalcemia was defined as iCa being <0.9 mmol/L, whereas hypercalcemia was defined as iCa being >1.35 mmol/L. Hyponatremia was defined as sodium ion concentration being <135 mmol/L, whereas hypernatremia was defined as sodium ion concentration being >145 mmol/L. Metabolic acidosis was defined as bicarbonate levels being <22 mmol/L, whereas metabolic alkalosis was defined as bicarbonate levels being >27 mmol/L.

### Statistical analysis

Enumeration data were analyzed using the chi-squared test. Normally distributed continuous variables were presented as mean ± standard deviation and compared using the *t* test or expressed as median (first and third quartile) and compared using the Mann–Whitney *U* test, as appropriate. Statistical analyses were performed with IBM SPSS version 21 software. A *P*-value of <0.05 was considered statistically significant.

## Results

### Patients’ characteristics

In total, 23 newborns (14 males) who collectively underwent 57 sessions of RCA-CRRT were included. At the time of CRRT initiation, the mean age was 10.1 ± 6.9 days (corrected for gestational age, 268 ± 24 days), and the mean body weight was 3.0 ± 0.7 kg (range, 0.95–4 kg). There were 25 (43.8%) sessions with prothrombin time >17 s and 46 (82.5%) sessions with activated partial thromboplastin time >47 s before treatment. Nine children died in the hospital, and the death was not directly attributed to CRRT. The demographic and clinical characteristics of the patients are described in Table 2.

**Table 2:** Clinical characteristics of patients.

Parameter	Value
*N*	23
Sessions, *n*	57
Age, days	10.1 ± 6.9
Correct gestational age, days	268.3 ± 24.7
Body weight, kg	3.0 ± 0.7
Sex, male	14
PRISM III score	26.27 ± 0.64
In-hospital mortality	9 (39.1%)
Cause of death	
Low cardiac output syndrome	2
Severe hypoxic ischemic encephalopathy	2
Necrotizing enterocolitis	1
Persistent pulmonary hypertension	1
Severe congenital diaphragmatic hernia	1
Severe hyperammonemia	2
28-day mortality	11 (47.8%)
Primary diagnosis	
Cardiac disease	10
Sepsis	2
Metabolic disease	3
Renal	2
Primary pulmonary	1
Intestinal disease	3
Others	1
Bleeding risk before CRRT	
Postoperative	13/23 (56.5%)
Intracranial hemorrhage	4/23 (17.4%)
Prothrombin time >17 s	25/57 (43.8%)
Activated partial thromboplastin time >46 s	46/57 (82.5%)
Platelets <100 × 10^9^	32/57 (56.1%)
Filter lifespan, h	31.54 ± 19.58
<24 h	22
Reasons for filter lifespan <24 h	
Ionized calcium *in vitro* >0.4 mmol/L	11
Catheter malfunction	10
Hypotension	1
>48 h	12
Reasons for disconnection	
Catheter malfunction	8
Discontinuation of treatment or death	11
Clotting	36
72 h filter change	2

Data are presented as mean ± SD (range) or percentage. Correct gestational age: gestational age + days since birth.

### Effectiveness

The mean filter lifespan was 31.54 ± 19.58 h (range, 3.3–72.5 h). The mean ionized calcium *in vitro* during RCA-CRRT was 0.37 ± 0.08 mmol/L. A total of 207 measurements of ionized calcium *in vitro* were obtained, of which 128 (61.8%) were <0.4 mmol/L. The distribution of filter lifespan and reasons for disconnection are shown in Table 2. After the treatment, creatinine levels decreased significantly but urea nitrogen and lactic acid levels did not (Table 3).

**Table 3:** Comparison of blood gas analysis and biochemical indices before and after RCA-CRRT.

	Before RCA-CRRT (*n* = 57)	After RCA-CRRT (*n* = 51)	*P* value
Urea nitrogen, mmol/L	8.82 ± 6.11	8.80 ± 8.18	0.987
Creatinine, mmol/L	92.96 ± 51.58	73.90 ± 27.20	0.032
Lactic acid, mmol/L	2.40 (1.05, 4.50)	1.80 (0.90, 3.00)	0.087
pH	7.37 ± 0.14	7.44 ± 0.11	0.001
<7.35	22	10	0.025
>7.45	19	22	0.198
Bicarbonate, mmol/L	25.70 ± 6.27	29.66 ± 4.95	0.000
<22 mmol/L	15	4	0.010
>27 mmol/L	23	37	0.001
Ionized calcium *in vivo*, mmol/L	1.18 ± 0.14	1.17 ± 0.13	0.692
<0.9 mmol/L	3	1	0.352
>1.35 mmol/L	4	4	0.578
T/iCa	2.00 ± 0.36	2.19 ± 0.40	0.056
>2.5	5/44 (11.4%)	5/35 (14.3%)	0.477
Ionized sodium, mmol/L	139.74 ± 6.38	139.85 ± 6.08	0.928
<135 mmol/L	13	8	0.246
>145 mmol/L	10	6	0.285

T/iCa, the ratio of total calcium to ionic calcium.

### Complications

After treatment, pH value, bicarbonate levels, and the incidence of bicarbonate levels being >27 mmol/L increased significantly, and the incidence of pH being <7.35 and bicarbonate levels being <22 mmol/L decreased significantly. No significant difference was noted in the concentration of ionized calcium *in vivo*, concentration of ionized sodium, the incidence of pH being >7.45, hypernatremia, hyponatremia, hypercalcemia, and hypocalcemia. Both T/iCa and the incidence of T/iCa being >2.5 increased, but the increase was not significant (Table 3).

### Influencing factors of CA

Thirty-five T/iCa measurements were obtained after treatment, and 22 measurements could not be obtained because of reasons such as child death or abandonment during CRRT. After CRRT, there were five sessions with T/iCa > 2.5 (CA group) and 30 sessions with T/iCa < 2.5 (NCA group). The molers of citrate per CiFR/BW was lower in the CA group than in the NCA group, but the difference was not significant. The weight was significantly higher in the CA group than in the NCA group, and the BFR/BW was significantly smaller in the CA group than in the NCA group. However, the two groups showed no significant difference in terms of age, corrected gestational age, PRISM-III score, or biochemical test findings, such as prothrombin time, activated partial thromboplastin time, total bilirubin, direct bilirubin, aspartate aminotransferase, alanine transaminase, GGT, lactic acid, and terminal parameters, such as CiFR/BFR and CaFR/BFR (QD + QF)/BW. The details in this regard are shown in Table 4.

**Table 4:** Comparison of CA and NCA group after RCA-CRRT.

Parameter	NCA group	CA group	*P* value
*N*	30	5	
Before RCA-CRRT			
PRISM III score	25.00 (24.75, 28.00)	27.00 (26.00, 28.00)	0.242
Age, days	8.80 ± 4.76	7.40 ± 2.51	0.646
Correct gestational age, days	274.4 ± 18.2	281.0 ± 2.2	0.454
Weight, kg	2.95 ± 0.41	3.64 ± 0.32	0.033
Before RCA-CRRT			
Prothrombin time, s	11.12 ± 8.81	12.76 ± 7.54	0.355
Activated partial thromboplastin time, s	69.50 ± 28.92	114.60 ± 62.17	0.166
Total bilirubin, μmol/L	137.83 ± 73.44	134.98 ± 45.51	0.904
Direct bilirubin, μmol/L	4.55 (0.00, 14.58)	3.50 (1.45, 21.05)	0.535
Aspartate aminotransferase, IU/L	55.00 (28.75, 138.00)	48.00 (39.90, 97.50)	0.906
Alanine transaminase, IU/L	27.78 ± 10.14	28.00 ± 9.01	0.976
Glutamyl transpeptidase, IU/L	80.72 ± 55.09	26.26 ± 2.80	0.095
Lactic acid, mmol/L	3.44 ± 2.10	4.91 ± 3.20	0.461
Final parameters			
BFR/BW, ml/min	4.07 ± 0.71	3.08 ± 0.08	0.027
CiFR/(60*BFR)	1.45 ± 0.14	1.37 ± 0.12	0.287
CaFR/(60*BFR)	0.117 ± 0.024	0.140 ± 0.029	0.200
(DFR + FFR)/BW, ml/h	55.1 ± 12.4	48.5 ± 11.4	0.551
Molers of citrate per CiFR/BW, mmol/kg/h	0.774 ± 0.185	0.637 ± 0.071	0.117
CRRT duration, h	25.8 ± 12.7	21.1 ± 6.1	0.575

CA, T/iCa > 2.5 at the end of RCA-CRRT; NCA, T/iCa < 2.5 at the end of RCA-CRRT; BFR, blood flow rate, ml/min; BW, body weight, kg; CiFR, flow rate of 4% sodium citrate, ml/h; CaFR, flow rate of 10% calcium gluconate, ml/h; DFR, dialysate flow rate, ml/h; FFR, filtrate flow rate, ml/h.

### Parameter adjustment

CiFR/BFR and CaFR/BFR were the only treatment settings to be significantly altered compared with initial treatment parameters. The details are shown in Table 1.

## Discussion

In this study, we analyzed the performance of RCA-CRRT in critically ill newborns. We found RCA to be safe and effective for CRRT anticoagulation in newborns. The risk of CA in neonates with RCA-CRRT was not significantly related to age and corrected gestational age, and the citrate metabolism capacity of neonates may not be worse than that of children or adults.

The requirements for CRRT anticoagulation are more stringent in neonates than in children and adults. It is well known that a slower blood flow rate is associated with a higher risk of clotting. Therefore, effective anticoagulation *in vitro* is extremely important to prolong filter life. In addition, neonatal CRRT is often complicated by high risk of bleeding. First, critically ill neonates are often associated with high risk of hemorrhage, which typically manifests as coagulation disorders, and premature infants are particularly vulnerable to hemorrhage due to immaturity of the brain, lung, and other organs. In this study, of the 57 sessions of RCA-CRRT, there were 25 (43.8%) sessions with prothrombin time >17 s and 46 (82.5%) sessions with activated partial thromboplastin time >47 s before treatment. At the first RCA-CRRT, the corrected gestational age of 6 (26.1%) out of 23 neonates was still <37 weeks. Second, due to the low blood volume of neonates, the pre-filling with red cell suspension and albumin before CRRT will significantly reduce the concentration of coagulation factors, and after CRRT, the extracorporeal blood is often not returned to the body, resulting in direct loss of coagulation factors. The blood is not returned because the minimum blood pump velocity of 1 ml/min is still too high, which may lead to cardiac deterioration or even cardiac failure in newborns. The average weight of newborns in the current study was 3 kg, and the extracorporeal circulation volume was about 1/3–1/4 of the newborns’ total blood volume. In addition, thrombocytopenia was observed in the CRRT sessions, which increases the risk of bleeding (17). The decrease in platelet count can be attributed to platelet activation and degranulation from exposure to the roller pump in the CRRT machine and by attaching with the microbubbles or dialysate (15). A hemofilter could contribute to thrombocytopenia during CRRT by either destruction or retention of platelets during passage (18). Therefore, a protocol with an exact anticoagulation effect *in vitro* and without affecting their own coagulation function *in vivo* is more suitable for neonatal CRRT.

RCA can maintain effective anticoagulation during CRRT in newborns. Ionized calcium *in vitro* concentration of 0.2–0.4 mmol/L was typically used as the anticoagulation target during RCA-CRRT (19–22). In this study, during RCA-CRRT, the mean ionized calcium *in vitro* concentration was 0.37 ± 0.08 mmol/L, and the ionized calcium *in vitro* concentration was <0.4 mmol/L in 61.8% sessions. The mean filter lifespan was 31.54 ± 19.58 h (range, 3.3–71.5 h), which was comparable to the filter lifespan of other neonatal CRRTs anticoagulated with heparin (14–51.1 h) (23, 24).

Excessive ionized calcium *in vitro* and poor catheter function are major factors limiting the filter lifespan of neonatal RCA-CRRT. In this study, there were 22 sessions with a filter lifespan of <24 h, of which 11 (50%) sessions had ionized calcium *in vitro* >0.4 mmol/L, 10 (45.5%) sessions were considered to be caused by poor catheter function, and the remaining 1 (4.5%) session was related to hypotension.

The CA risk associated with RCA-CRRT in neonates is not higher than it is in children or adults and is not significantly related to the gestational age. Notably, CA is the most concerning complication of neonatal RCA-CRRT, and neonatologists often worry that the liver and other organs of newborns are immature, which will lead to insufficient citrate metabolism. In this study, there was no significant difference in the incidence of CA before and after the treatment. After RCA-CRRT, the incidence of CA was 14.3%, which was significantly lower than that reported by Persic et al. in their study on neonatal and infant RCA-CRRT (25%) (14). Compared with the NCA group, in the CA group, the body weight was significantly higher and the BFR/BW value was significantly lower, and the molers of citrate per CiFR/BW value was lower but without statistical significance. However, this does not suggest that children with higher body weights and smaller molers of citrate per CiFR/BW values are more likely to develop CA. The significant difference in body weight is more likely to be related to the bias caused by the small number of CA cases. Conversely, in the CA group, to reduce the risk or degree of CA, we further reduced BFR/BW, thereby resulting in a smaller molers of citrate per CiFR/BW value. In other words, as CA had already occurred, we further reduced CiFR to reduce the degree of CA or even avoid CA. In addition, there was no significant difference in age (days), corrected gestational age, molers of citrate per CiFR/BW, prothrombin time, activated partial thromboplastin time, aspartate aminotransferase, alanine transaminase, lactic acid, and other indicators between the CA and NCA group. This indicates that the occurrence of CA in neonatal RCA-CRRT may not be associated with whether the birth was preterm or not and the degree of preterm birth. The average molers of citrate per CiFR/BW in this group was 0.79 mmol/kg/h, which was similar to the minimum predicted accumulation concentration reported by Persic et al. (14) (0.78–1.7 mmol/L).

## Limitation

The current study has some limitations. Given its retrospective design, some data are missing. In addition, the sample size is small and may be subject to statistical bias; however, to our knowledge, this is the largest sample size in a neonatal RCA-CRRT study to date.

## Conclusion

RCA is safe and effective for neonatal CRRT anticoagulation. Neonates with RCA-CRRT were not at a significantly higher risk of developing CA than children or adults. The risk of CA showed no significant correlation with gestational age or corrected gestational age.

## Data Availability

The original contributions presented in the study are included in the article/Supplementary Material, further inquiries can be directed to the corresponding author.
